# tRNA-derived small RNA 3’ tRF-Ala CGC obstructs NK cytotoxicity via cleavage of membrane protein MICA in colorectal cancer

**DOI:** 10.3389/fimmu.2025.1620550

**Published:** 2025-07-14

**Authors:** Jing Zhang, Chunlin Ou, Xin Li, Li Fu, Qizhi Luo, Jie Wang, Yizhou Zou

**Affiliations:** ^1^ Department of Immunology, Xiangya School of Basic Medicine, Central South University, Changsha, Hunan, China; ^2^ Department of Pathology, Xiangya Hospital, Central South University, Changsha, Hunan, China

**Keywords:** tsRNA, 3’ tRF-AlaCGC, MICA, ADAM10, NK cells, immune evasion

## Abstract

**Background:**

Immune escape remains a major challenge in cancer immunotherapy. Transfer RNA (tRNA)-derived small RNA (tsRNA) represents a novel class of non-coding RNAs generated from tRNA cleavage, regulating gene expression at transcriptional and translational levels. These tsRNAs exhibit diverse biological functions, including immune modulation, metabolic disorders, and cell death. Despite their critical involvement in tumor progression, the role of tsRNAs in Natural killer (NK) cells related to immune escape within colorectal cancer (CRC) has not been revealed yet.

**Methods:**

High-throughput sequencing and the tRFexplorer database were utilized to compare the profiles of CRC and normal tissues. Techniques such as RT-qPCR, western blotting, and flow cytometry were employed to assess gene and protein expression. The Cell Counting Kit-8 assay, colony formation assay, and apoptosis analysis were used to evaluate tumor heterogeneity. Differential gene expression between the tRF-3021a inhibitor and negative control (NC) in HCT116 cells was quantified and characterized using RNA sequencing.

**Results:**

We identified 3’ tRF-AlaCGC (tRF-3021a) as significantly upregulated in CRC tissues. Major histocompatibility complex class I related chain A (MICA) is an important and stress-induced ligand of the natural killer group 2 member D receptor (NKG2D) that is expressed in various cancer cells. MICA undergoes post-translational modifications that regulate their expression as they are called membrane-bound MICA (mMICA) at the cancer cell surface. mMICA is a ligand that induces the activation of NK cells. Proteolytic cleavage of mMICA by A Disintegrin Metalloproteinase Domains (ADAMs) is the underlying mechanism in CRC. Mechanistically, tRF-3021a promotes proteolytic cleavage of mMICA by upregulating ADAM10, generating soluble MICA (sMICA). Elevated sMICA acts as a decoy ligand for NKG2D receptors on NK cells, impairing cytotoxicity and facilitating immune escape. Functional assays confirmed that tRF-3021a knockdown enhances NK cell-mediated CRC cell killing, while overexpression promotes CRC proliferation and inhibits apoptosis. Clinically, tRF-3021a is elevated in CRC tissues, serum exosomes, and cell lines, cleaved by ANG, demonstrating diagnostic potential. *In vivo*, experiments provided further evidence that inhibition of tRF-3021a reduce tumorigenicity.

**Conclusion:**

Our findings reveal tRF-3021a as a novel biomarker and therapeutic target for CRC immunotherapy.

## 1 Introduction

Colorectal cancer (CRC) maintains its position as the third most commonly diagnosed malignancy and the second leading cause of cancer-associated deaths worldwide (1). The insidious nature of CRC progression is characterized by non-specific clinical manifestations during early disease stages, frequently resulting in delayed diagnosis at advanced metastatic phases (2). However, given the inherent metastatic propensity of colorectal malignancies, radical surgical intervention often proves therapeutically inadequate for metastatic CRC. Since metastatic colorectal cancer is not easily resectable, systemic therapy (cytotoxic chemotherapy, biologic therapy such as growth factor antibodies, targeted therapy, immunotherapy, and combination therapy) is used primarily to improve the prognosis of patients with colorectal cancer. Notwithstanding these advances significantly, the critical need persists for novel molecular targets and validated biomarkers to optimize therapeutic efficacy (2).

Cancer immunotherapy is an active or passive way to make the body produce a tumor-specific immune response, exert its function of inhibiting and killing tumor cells, and has the advantages of specific and efficient treatment to prevent the body from harm (3). Immunotherapy represents a paradigm-shifting therapeutic modality that orchestrates tumor-specific immune responses through active immunization or passive immune potentiation, harnessing host immunity to eradicate malignant cells while minimizing off-target toxicity selectively (4). Immunotherapy is not to kill cancer cells, but to mobilize the body to recognize the tumor immune cells, improve the body’s immune system combat ability, rely on them to indirectly kill and control cancer, and have side effects that are small, safe, and effective directly (4). Distinct from conventional cytotoxic approaches, this strategy reprograms immune surveillance mechanisms by enhancing immune cell recognition capacity and reinvigorating antitumor effector functions, thereby achieving sustained tumor control with improved safety profiles. However, immune evasion mechanisms developed during tumor immunoediting continue to compromise therapeutic responses, with a substantial proportion of patients exhibiting primary or acquired resistance to current immunotherapies. Consequently, identifying novel immunotherapeutic targets and developing strategies to counteract immunosuppressive tumor microenvironments remain critical methods in oncology research (5).

Natural killer cells (NK cells) are innate cytotoxic lymphoid cells, which are essential for tumor cell clearance and cancer immune surveillance through dynamic receptor-ligand interactions (6). The interaction of NK cell receptors with target cells influences disease progression (7). The activation of NK cells is influenced by the involvement of different receptor-ligands in the regulation of activation and inhibition signals (8). NK cells are able to kill abnormal cells, including tumor cells and virus-infected cells, because the process activates the natural killer Group 2 member D receptor (NKG2D) (also known as killer cell lectin like receptor K1, KLRK1), which regulates the immune response mechanism of NK cells after receiving ligand signals (8). This pivotal immunoreceptor recognizes eight distinct ligands (MICA, MICB, and ULBP/RAET1 family members), orchestrating immune recognition through direct tumor cell engagement (6).

In tumor immune surveillance, the NKG2D axis maintains a delicate equilibrium between immune activation and inhibition during cancer immunoediting (9). On the one hand, the activation of the NKG2D receptor can enhance the killing ability of NK cells (7). Membrane-bound MICA (mMICA) expression on malignant cells correlates positively with NK cell-mediated cytotoxicity, suggesting therapeutic potential for mMICA upregulation strategies (10). When NK cells recognize mMICA on the surface of tumor cells through NKG2D and promote a cytotoxic reaction, the expression level of mMICA on the surface of tumor cells may determine the anti-tumor effect and activate the killing function of NK cells. Therefore, overexpression of mMICA may be an effective strategy to limit tumor progression (11). On the other hand, dysregulation of NKG2D ligand expression may be the main source of cellular immunogenic dysregulation. Paradoxically, cancer cells employ multiple immune evasion mechanisms, including proteolytic shedding of MICA by ADAM metallopeptidases. The hydrolytic cleavage of MICA membrane proteins by adisintegrin metalloproteinase domains (ADAMs) and matrix metalloproteinases (MMPs) is a potential immune escape mechanism. ADAMs and MMPs families can generate soluble MICA (sMICA) (12). sMICA-mediated NKG2D internalization induces receptor degradation and functional NK cell paralysis, facilitating immune escape (9).

Non-coding RNAs (ncRNAs), including microRNAs (miRNAs), long non-coding RNAs (lncRNAs), and circular RNAs (circRNAs), have emerged as crucial regulators of tumorigenesis and immune modulation. Our previous investigations revealed extensive ncRNA-mediated regulation of the MICA/NKG2D axis, modulating NK cell activity through post-transcriptional control mechanisms (11, 13). For example, hypoxia can induce the expression of circ_0000977 and sMICA in Panc-1 cells. circ_0000977/miR-153 axis can regulate HIF1a and ADAM10, dampening NK cell cytotoxicity through sMICA overproduction (9). Similarly, MICA targets miRNA clusters (miR-17, miR-93, miR-20a) that inhibit BCL11B expression. These miRNAs bind to the BCL11B 3’-UTR, causing BCL11B-mediated downregulation of MICA/B. Consequently, reduced NKG2D recognition leads to diminished elimination by NK cells (11).

In particular, some ncRNAs influence the development of various tumors, including CRC, and may serve as cancer biomarkers or therapeutic targets (14). With advances in RNA sequencing, a novel transfer RNA (tRNA) small RNAs (tsRNAs) (13–48 nt) has emerged as key players in carcinogenesis and treatment resistance. These tsRNAs are generated through stress-induced tRNA cleavage and include two major subtypes: tRNA fragments (tRFs) and tRNA halves (tiRNAs) (15). tsRNAs originate from ncRNAs generated by ribonucleases (RNases) such as angiogenin (ANG) or Dicer under stress conditions (hypoxia, starvation, viral infection, arsenite, heat shock, or heavy metal-induced cellular stress/toxicity conditions). These tsRNAs regulate gene expression at transcriptional and translational levels through mechanisms similar to miRNAs (16). Studies have shown that tRFs and tiRNAs are directly involved in RNA silencing, tsRNAs can form a complex with Argonaute (AGO) protein (17). tsRNAs can interact with and inhibit the expression of the target gene by binding to the 3’-untranslated region (3’-UTR) of the target mRNA, and can also compete with mRNA to bind RBP protein to regulate mRNA function (18). involved in cell proliferation, migration, invasion and other processes (19).

Recent studies have shown that dysregulation of tsRNAs in colorectal cancer is closely related to its occurrence and progression, highlighting its potential value in early diagnosis, prognosis assessment and treatment strategies (20). In CRC pathogenesis, tsRNA dysregulation demonstrates diagnostic, prognostic, and therapeutic relevance. 5’ tiRNA-His-GTG is up-regulated in colorectal cancer tissues that can resist apoptosis. Mechanically, the response process of 5’ tiRNA-His-GTG to the tumor hypoxic microenvironment, regulated by the HIF1α/ANG axis, reveals a specific pathway of 5’ tiRNA-His-GTG involved in colorectal cancer progression and provides clues for the design of new colorectal cancer therapeutic targets (21). Moreover, tRF/miR-1280 is derived from the 17 nt fragment of tRNA Leu and pre-miRNA, which affects Notch signaling and is related to cancer stem cell-like function. The expression of tRF/miR-1280 was decreased in human colorectal specimens, and overexpression of tRF/miR-1280 could reduce cell proliferation. Notch ligand JAG2, as a direct target of tRF/miR-1280, can reduce tumor formation and metastasis. Notably, tRF/miR-1280 mediates Notch inactivation signaling, including direct transcriptional suppression of Gata1/3 and miR-200b genes (22). In addition, the expression of tRF-16-7X9PN5D was significantly down-regulated in radiation-resistant CRC cells. tRF-16-7X9PN5D can promote the proliferation, migration, invasion, and radiation resistance of CRC cells by targeting MKNK1 and regulate the phosphorylation of eIF4E (23). This discovery provides a potential target for developing new therapeutic strategies for CRC.

Despite these advances, the functional interplay between tsRNAs and NK cell-mediated immune surveillance remains unexplored. This critical mechanism motivates our investigation into tsRNA-regulated immune evasion mechanisms in colorectal carcinogenesis, specifically focusing on tRF-3021a/ADAM10/MICA/NKG2D axis modulation. We will conduct an meaningful analysis of this study on the immune escape mechanism that inhibits NK cell function.

## 2 Methods

### 2.1 Clinical samples

The study was approved by the EC of Guizhou Provincial People’s Hospital Ethical Review Approval. From 2021 to 2024, we collected serum and tissue samples from patients with CRC. All samples were stored at -80°C after centrifugation until further processing. Human colorectal cancer tissues and adjacent tissues were collected from patients who had not received any radiotherapy or chemotherapy. All patients were anonymized, informed of the purpose and content of the study, and signed an informed consent form.

### 2.2 Cell culture and cell transfection

Human colon mucosal epithelial NCM460 cells and 3 colorectal cell lines (HCT116, HCT8, and SW620) were purchased from ATCC (Manassas, VA, USA). To obtain mRNA expression data, the cell lines were cultured in RPMI 1640 medium or DMEM high glucose supplemented with 10% fetal bovine serum, and 10% penicillin-streptomycin solution in a humidified 5% CO_2_ incubator at 37°C. Cells were seeded into 6-well or 12-well plates 24 h before transfection. Cell transfection reagent was performed with Lipofectamine 3000 reagent (Invitrogen, USA). Inhibitor NC, mimics NC, tRF-3021a inhibitor or tRF-3021a mimics, 36h after transfection, RNA extraction was performed by TRIzol method and flow cytometry detected cell apoptosis and protein extraction was performed by cells collection 48h after transfection. The tRF-3021a inhibitor and mimics were gained from RiboBio (Guangzhou, China). SiRNA-ANG and siRNA NC were produced from Tsingke (Beijing, China). tsRNA Inhibitor and siRNAs-dependent transfection reagent: It needs to form a complex with cationic liposomes (such as Lipofectamine 3000), encapsulate the nucleic acid, and then enter the cell through endocytosis.

### 2.3 RNA isolation, cDNA synthesis, and quantitative real-time PCR

The total RNA of cells was isolated with TRIzol (CWBIO, China). Then, RNA was quantified using The Evo M-MLV RT Premix (Code. AG11603, Accurate Biotechnology, Hunan, China), which was used for mRNA reverse transcription in a 20 μl reaction volume according to the manufacturer’s protocol. The miRNA 1st Strand cDNA Synthesis Kit (Code. AG11745, Accurate) was used for tsRNAs reverse transcription in a 20 μl reaction volume and then diluted to 200 μl. The cDNA amplification was performed by RT-qPCR using SYBR Green Pro Taq HS Premix (Code. AG11702, Accurate). GAPDH was used as the internal control for mRNA, and U6 was used as the internal control for tsRNAs. The expressions of genes related to internal controls were detected using the 2−ΔΔCt method, and each assay was repeated three times. The sequence information of all primers is listed in **Supplementary Table 1**.

### 2.4 NK cell cytotoxic activity

The NK cell line (NKL, provided by M. J. Robertson, Indiana University School of Medicine, Indianapolis) was maintained in RPMI-1640 medium supplemented with 15% fetal bovine serum (FBS) and 10 ng/mL recombinant IL-2 (Sino Biological Inc.), cultured at 37°C in a 5% CO_2_ atmosphere. For cytotoxicity assays, HCT116 cells and SW620 cells were washed with sterile PBS and centrifuged at 800 rpm for 5 minutes. The cell pellet was resuspended in sterile PBS at a density of 1×10^6^ cells/mL and stained with 200 nM carboxyfluorescein succinimidyl ester (CFSE; Cat. C34554, Thermo Fisher Scientific) in PBS for 20 minutes at 37°C. After three PBS washes, CFSE-labeled target cells were plated in 96-well plates at 5×10^4^ cells/well (100 μL/well). 1×10^5^ NKL cells/well (100 μL/well) effector cells were added to target cells at specified effector-to-target (E: T) ratios (5:1) in a final volume of 200 μL complete medium. Co-cultures were incubated at 37°C for 5 hours. Cells were subsequently centrifuged at 2000 rpm for 2 minutes, washed twice with PBS containing 2% FBS, and stained with 7-amino actinomycin D (7-AAD; BD Pharmingen, Cat. 51-68981E) at room temperature for 10 minutes. Cell viability was assessed by flow cytometry, with CFSE^+^ target cells analyzed for 7-AAD incorporation to quantify apoptosis.

### 2.5 Flow cytometry analysis

NCM460, HCT116, HCT8, and SW620 cells were washed twice with PBS and resuspended in PBS. A volume of 100 μL of these cells was stained with MICA antibody (Production in our laboratory) and 1 μL second antibody is PE Goat anti-mouse IgG Antibody (Biolegend, USA). Apoptosis assay was measured by flow cytometry using the Annexin V-FITC/PI kit (Apexbio, USA).

### 2.6 Detection of soluble MICA by Enzyme-linked immunosorbent assay

Supernatants from cells cultured for 2 days were analyzed using ElaBoXTM Human MICA ELISA Kit (Cat. SEKH-0241, Solarbio).

### 2.7 Western blot

Cultured cells were washed with PBS and lysed for 30 min on ice with RIPA buffer containing 1% protease inhibitor. Protein concentrations were determined by using a BCA kit (Thermo Fisher, USA). Equal amounts of proteins were then resolved on SDS-PAGE gel, transferred to PVDF membrane, and incubated with ADAM10 antibody (1:1000, Bioworld Technology, China) and GAPDH antibody (1:1000, Proteintech, China) overnight at 4°C. On the following day, the secondary antibody was incubated for 1 h to interact with the primary antibody.

### 2.8 Cell proliferation

Cell proliferation was assessed using the Cell Counting Kit-8 (Cat. K1018, Apexbio, USA) at time points of 0h, 24h, 48h, 72h, and 96h. A total of 1500 cells per well were seeded in a 96-well culture plate.

### 2.9 Colony formation assay

For colony formation, 500 cells were seeded into a six-well culture plate and cultured for 14 days. The colonies were then stained with Crystal Violet (Cat No. C0121, Beyotime, China).

### 2.10 RNA sequencing and bioinformatics analysis

RNA sequencing was performed at Oebiotech. The tRF Explorer database, which provides the expression profiles of tRFs in various tumor tissues (https://trfexplorer.cloud/, February 2025), was utilized for this study. Data from colorectal cancer (CRC) and normal colon tissues were obtained from The Cancer Genome Atlas (TCGA). Differential gene expression analysis was conducted using the Wilcoxon rank-sum test in R software. The expression level of ADAM10 protein in CRC was assessed using immunohistochemistry (IHC) images from The Human Protein Atlas (HPA). The diagnostic value of ADAM10 and MICA in CRC was evaluated through receiver operating characteristic (ROC) curve analysis using TCGA datasets. An area under the curve (AUC) close to 1 indicates an improved diagnostic performance. AUC values greater than 0.9 were considered to have high diagnostic value, values between 0.9 and 0.7 were considered to have moderate diagnostic value, and AUC values between 0.7 and 0.5 were considered to have low diagnostic value.

### 2.11 *In vivo* animal model

All animal procedures were approved by the Experimental Animal Management (Ethics) Committee of Hunan SJA Laboratory Animal Co., Ltd (Approval No. SJA202501501). Eight female C57BL/6J mice (6–8 weeks old; Hunan SJA Laboratory Animal Co., Ltd.) were housed under specific pathogen-free conditions (22 ± 1°C, 50 ± 10% humidity, 12-h light/dark cycle). MC38 cells in logarithmic growth phase were harvested by enzymatic digestion and resuspended in sterile PBS at 2×10^6^ cells/100 μL. Under aseptic conditions, each mouse received a 100 μL subcutaneous injection of the cell suspension into the right axillary region (Day 0). When tumor volumes calculated as width² × length × 0.5, mice were randomized into two groups (n=4/group): Negative control (NC): 10 nmol scrambled antagomir. Treatment group: 10 nmol tRF-3021a antagomir (Genepharma, Shanghai). All antagomirs (cholesterol-modified for direct membrane penetration, no transfection reagent required) were administered via intratumoral injection every 72 h (Days 7, 10, and 13 post-implantation). Tumor dimensions and body weights were recorded every 48 h using digital calipers and analytical balances. On Day 17 post-implantation, mice were humanely euthanized. Excised tumors were immediately photographed, weighed.

### 2.12 Statistical analysis

Gene Ontology (GO) and Kyoto Encyclopedia of Genes and Genomes (KEGG) pathway enrichment analyses were conducted using R software (version 4.2.1) for differential expression analysis, with visualizations generated using ggplot2 (version 3.4.4). GraphPad Prism (v.9.5, USA) was used to perform statistical comparisons between different groups. For normally distributed data, the Student’s t-test was applied. Statistical significance was indicated as follows: *p < 0.05, **p < 0.01, ***p < 0.001.

## 3 Results

### 3.1 tRF-3021a is significantly upregulated in CRC

**Figure 1:**
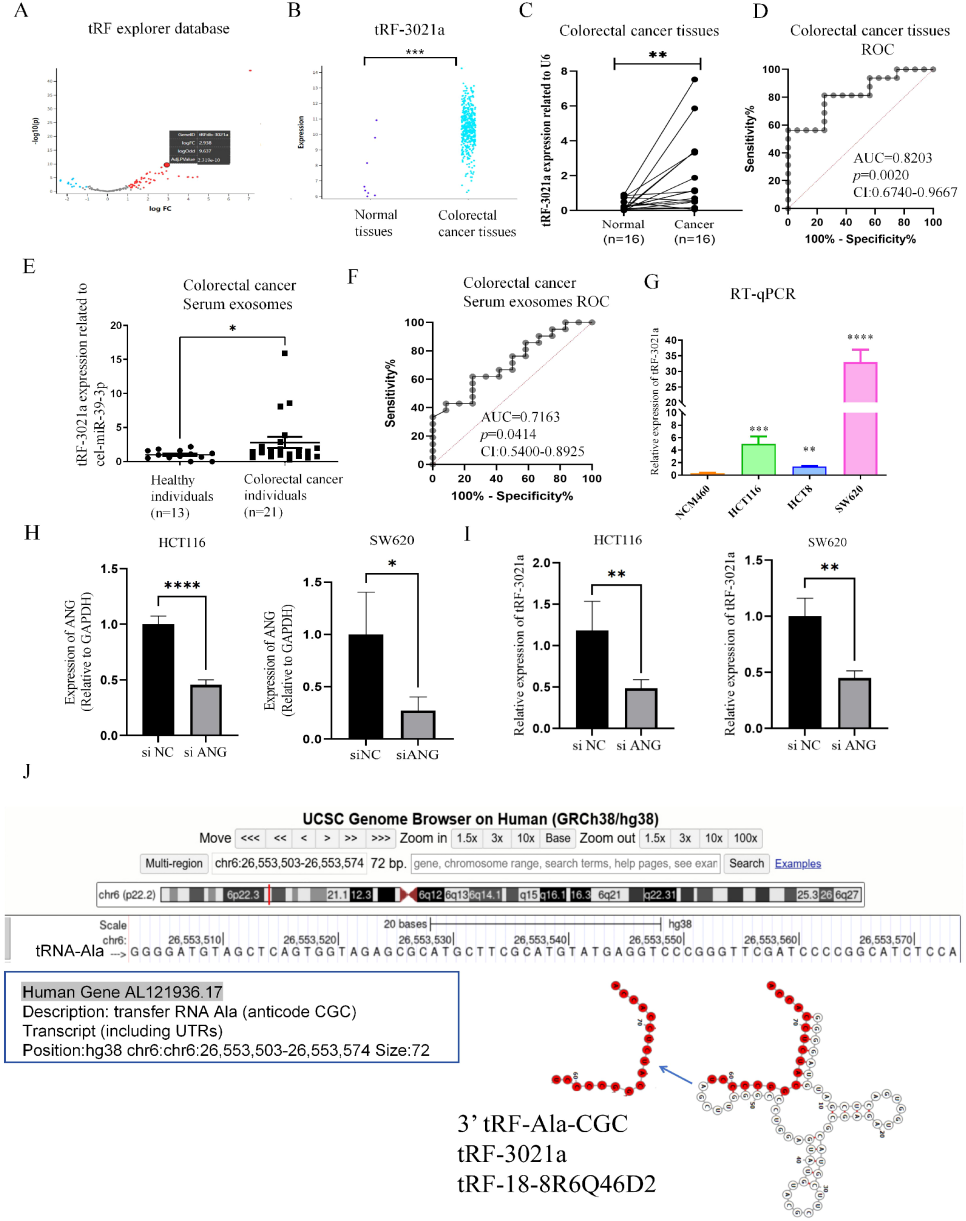
tRF-3021a is associated with colorectal cancer progression through ANG-mediated tRNA-Ala-CGC splicing. **(A)** Volcano plot illustrating differentially expressed tsRNAs between CRC and normal tissues in the tRF explorer database, showing significant overexpression of tRF-3021a in tumor tissues. **(B)** Comparative analysis confirming elevated tRF-3021a expression in CRC tissues versus normal tissues. **(C)** RT-qPCR validation of upregulated tRF-3021a levels in 16 paired CRC tissues compared with adjacent normal tissues. **(D)** ROC curve analysis evaluating the diagnostic values of tRF-3021a in CRC patient tissue samples. **(E)** Enhanced tRF-3021a expression detected by RT-qPCR in plasma exosomes from 21 CRC patients versus 13 healthy controls. **(F)** ROC analysis demonstrating the diagnostic potential of exosomal tRF-3021a. **(G)** RT-qPCR showing elevated tRF-3021a levels in CRC cell lines relative to normal colon mucosal epithelial NCM460 cells. **(H)** Expression of ANG in CRC cells with ANG knockdown. **(I)** Reduced tRF-3021a production following ANG knockdown, indicating ANG-dependent biogenesis. **(J)** UCSC genome browser visualization of tRF-3021a characteristics. *P < 0.05, **P < 0.01, ***P < 0.001, ****P < 0.0001.

Firstly, to explore and characterize differential tsRNAs expression in colorectal normal tissues and CRC, tRF and tiRNA sequencing was performed on tRF explorer database. Aberrantly expressed tsRNAs in human CRC may be potential diagnostic biomarkers of therapeutic targets (22). After considering the abundance of tsRNAs, statistical significance (*p* < 0.05), and the magnitude of differential expression (|log2FC| > 1.5), we followed the naming conventions from the tRF Explorer database, OncotRF, and tRFdb. Specifically, the 3’ tRNA-AlaCGC was named tRF-3021a and tRF-18-8R6Q46D2. Among these, tRF-3021a was selected for further investigation. The expression of tRF-3021a was significantly upregulated in CRC tissues (|log2FC| = 2.938, *p* < 0.05). (**Figures 1A, B**). As shown in **Figure 1C**, tRF-3021a was validated by RT-qPCR, which was significantly higher in 16 CRC tissues compared to paired normal tissues (**Figure 1D**). To evaluate the diagnostic potential of tRF-3021a in distinguishing CRC patients from healthy controls, receiver operating characteristic (ROC) curve analysis was performed. The Area Under Curve (AUC) for tRF-3021a was 0.8203 (95% confidence internal (CI): 0.6740-0.9667; *p*=0.0020). Additionally, this result is consistent with emerging evidence suggesting that tsRNAs exhibit remarkable stability in various biofluids, positioning them as promising candidates for liquid biopsy applications. Notably, tsRNAs, a novel class of circulating ncRNAs, maintain structural integrity and are abundance in systemic circulation via exosome-mediated transport, making them ideal targets for non-invasive cancer detection (24). These tsRNAs can be released into body fluids, such as serum, in the form of exosomes, serving as biomarkers for liquid biopsy (24). Furthermore, the clinical relevance of these molecules extends beyond diagnostic applications, as recent studies indicate that exosomes encapsulated tsRNAs can modulate immune cell functions through intercellular communication. Specifically, these regulatory ncRNAs may facilitate tumor immune evasion by altering immune response pathways, highlighting their dual role as diagnostic markers and functional mediators in oncogenesis (25).

Based on these insights, we specifically investigated the expression profile of plasma-derived exosomal tRFs in our patient cohort, given their established presence in circulating exosomes and potential clinical utility in cancer diagnostics. Considering the presence of tRFs in plasma exosomes, we analyzed expression level of tRFs in patient plasma-derived exosomes. Thus, we established a cohort by collecting plasma from patients with the healthy (n=13) and CRC patients (n=21). Then, we extracted exosomes from serum and used cel-miR-39 from nematode as a reference to detect the expression of tRF-3021a in serum exosomes from colorectal cancer samples and healthy human serum exosomes by RT-qPCR. Interestingly, the expression level of tRF-3021a was also significantly higher in the CRC patients serum exosomes than healthy serum exosomes (**Figure 1E**). Then, as shown in **Figure 1F** ROC analysis was performed, the AUC was 0.7163 (95% confidence internal (CI) [0.5400-0.8925], *p*=0.0414). RT-qPCR results showed that the expression of tRF-3021a in CRC cells (HCT116, HCT8, SW620) was higher than in normal colon cells (NCM460) (**Figure 1G**). Subsequently, we further explored the generation of tRF-3021a. Angiopoietin (ANG) is a member of the RNase A superfamily also known as RNase5 that has relatively weak ribonucleic acid decomposition activity (18). This protein is an effective mediator for the formation of new blood vessels, and this protein induces angiogenesis after binding to actin on the surface of endothelial cells (21). This protein also accumulates in the nucleolus, where it stimulates ribosomal transcription. Under stress conditions, the protein translocates to the cytosol, where it hydrolyzes cellular tRNA and affects protein synthesis. Knockdown of ANG results in an ability for tsRNA generation. To further search that tRF-3021a originated from tRNA Ala, ANG was knocked down in HCT116 and SW620 cells, respectively (**Figure 1H**). As a result, the relative expression of tRF-3021a were significantly decreased after ANG knockdown (**Figure 1I**). tRF-3021a was a potential CRC biomarker, originated from Ala-tRNA and was cleaved by ANG. As shown in **Figure 1J**, 3’ tRF-Ala-CGC/tRF-3021a, located at chromosome 6 (26,553,503-26,553,574) and a length of 72 bp (http://genome.ucsc.edu/). In the tRFdb (http://genome.bioch.virginia.edu/trfdb/search.php), the molecule of tRF-3021a belong to a class of 18nt small RNAs and the sequence is 5’-TCCCCGGCATCTCCACCA-3’. The fragments matched perfectly to the 3’ end of mature tRNA-Ala-CGC and cleavage site is located on the T-loop in the sequence.

### 3.2 tRF-3021a attenuates NK cell-mediated cytotoxicity against colorectal cancer cells

**Figure 2:**
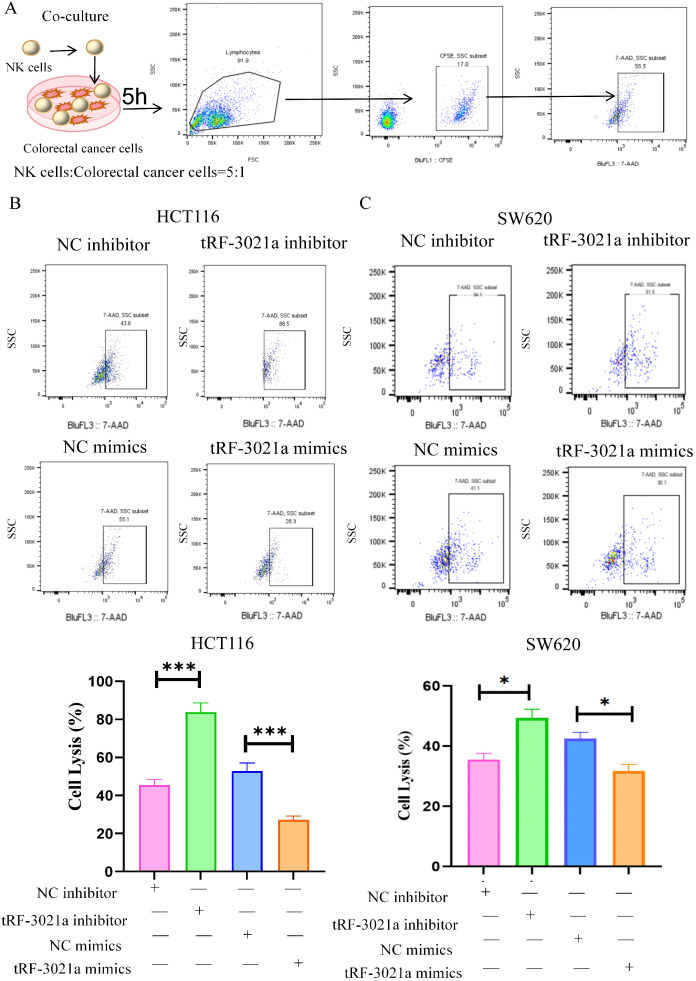
tRF-3021a reduced the sensitivity of CRC cells to NK cell killing. **(A)** Flowchart for the killing of NK cells and representative flow cytometry plots illustrate that were transfected with tRF-3021a inhibitor, mimics or control for 48h in CRC cells co-cultured (E: T=5:1 for 5h) and flow cytometry gating strategy. **(B, C)** NK cell cytotoxicity against tRF-3021a-overexpressing CRC cells and enhanced NK-mediated killing in tRF-3021a-knockdown models. Unpaired two-tailed t-test and one-way ANOVA were used for statistical analysis. *p < 0.05, ***p < 0.001.

Given the upregulated expression of tRF-3021a in colorectal cancer cells, we investigated its regulatory role in modulating CRC susceptibility to NK cell-mediated cytotoxicity. Through transient transfection with synthetic tRF-3021a mimics and inhibitors, along with establishment of stable gain/loss-of-function models in HCT116 and SW620 cell lines (expression validation shown in **Supplementary Figure S1**), we systematically evaluated tRF-3021a’ s immunomodulatory potential. We investigated the impact of tRF-3021a on NK cell-mediated anti-tumor immune responses against CRC cells. CRC cells (HCT116 and SW620) were transfected with tRF-3021a inhibitor and mimics, or NC for 48h. We stained HCT116 cells and SW620 cells with CFSE antibody as the target cells, which co-cultured with activated human NK cells for an additional 5h at an effector-to-target (E: T=5:1) ratio. Subsequent 7-AAD staining coupled with flow cytometric analysis (gating strategy detailed in **Figure 2A**) revealed altered cytotoxic responses. Notably, tRF-3021a overexpression significantly attenuated NK cell-induced tumor cell death (*p*<0.01), whereas its knockdown conversely enhanced CRC cell susceptibility to NK-mediated killing across both cellular models (**Figures 2B, C**).

### 3.3 Transcriptomic profiling revealed that tRF-3021a impairs NK cell immunosurveillance in colorectal cancer by modulating the ADAM10/MICA axis

**Figure 3:**
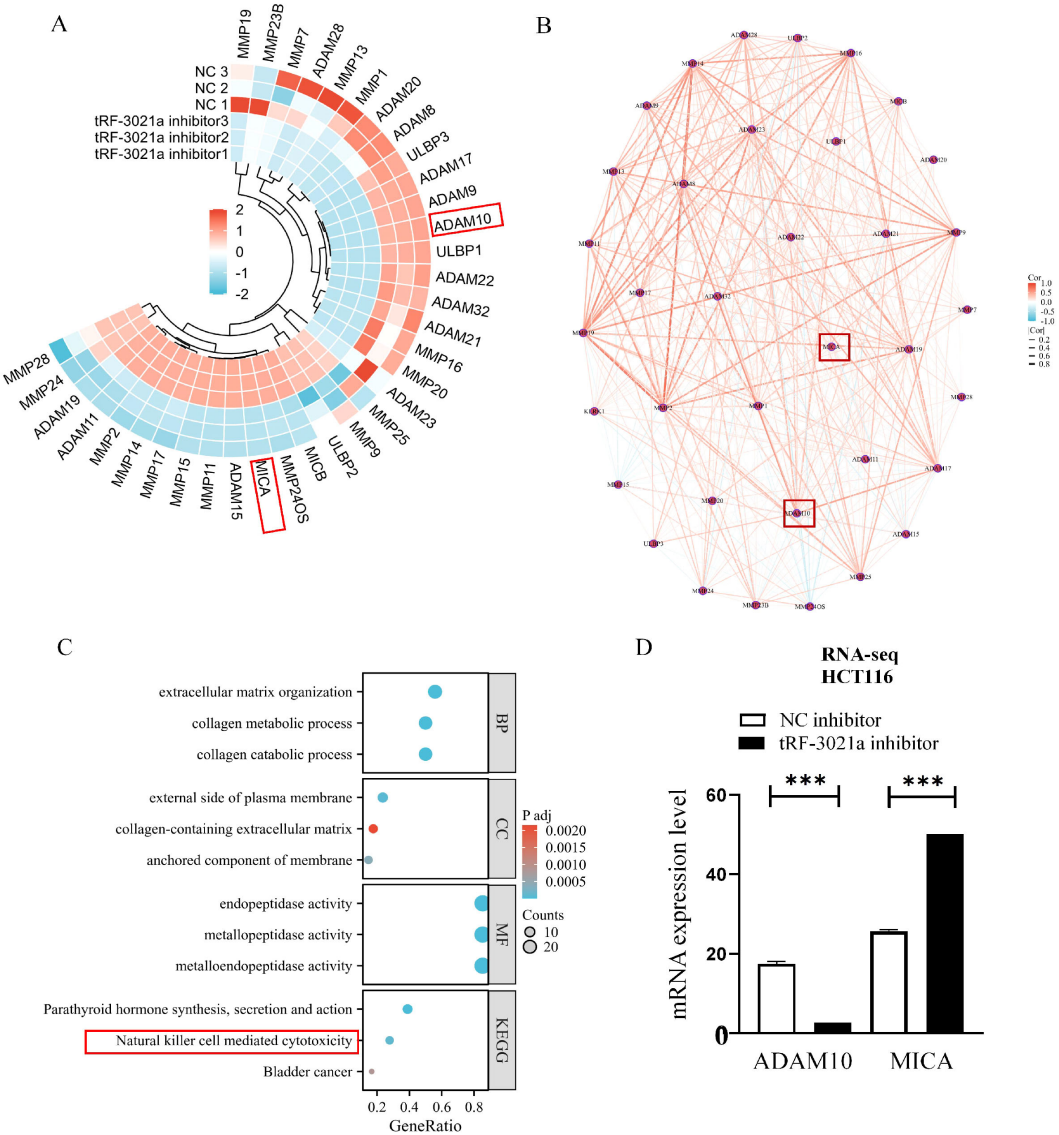
Transcriptomic profiling reveals tRF-3021a-mediated regulation of NK cell-related pathways in HCT116 cells. **(A)** Heatmap for NK cell related genes FPKM values in NC inhibitor groups and tRF-3021a inhibitor. **(B)** Protein-protein interaction network analysis of differentially expressed immunomodulatory genes. **(C)**GO/KEGG pathway enrichment of DEGs highlighting extracellular matrix organization (GO:0030198) and natural killer cell-mediated cytotoxicity (hsa04650). **(D)** RNA-seq analysis showing inverse regulation of ADAM10 and MICA expression in HCT116 cells. ***P < 0.001.

In our previous studies found that NK cell cytotoxicity is governed by the MICA/NKG2D immunological synapse, particularly through ADAM10-mediated proteolytic cleavage of membrane-bound MICA into immunosuppressive soluble forms (sMICA) (12). Therefore, we focused on tRF-3021a’s regulatory role in this axis. RNA sequencing of tRF-3021a-inhibited HCT116 cells (48h post-transfection) revealed significant alterations in ADAM family members and NK cell ligand expression. Heatmap analysis of differentially expressed genes (DEGs) encompassing NK cell ligands, ADAM proteases, and MMP family members demonstrated coordinated downregulation of ADAM10 concurrent with MICA upregulation (**Figure 3A**). Protein-protein interaction network modeling identified functional clustering among differential expressed genes (DEGs) (**Figure 3B**), with KEGG pathway enrichment analysis highlighting critical biological processes: extracellular matrix remodeling (GO:0030198, GO:0030574), plasma membrane protease activity (GO:0004222, GO:0008237), and most notably, natural killer cell-mediated cytotoxicity (hsa04650) (**Figure 3C**). Mechanistic validation confirmed that tRF-3021a knockdown significantly suppressed ADAM10 transcription (*p*<0.01) while elevating MICA mRNA levels (*p*<0.05) in HCT116 cells (**Figure 3D**).

### 3.4 tRF-3021a activates ADAM10 to drive immune escape in colorectal cancer

**Figure 4:**
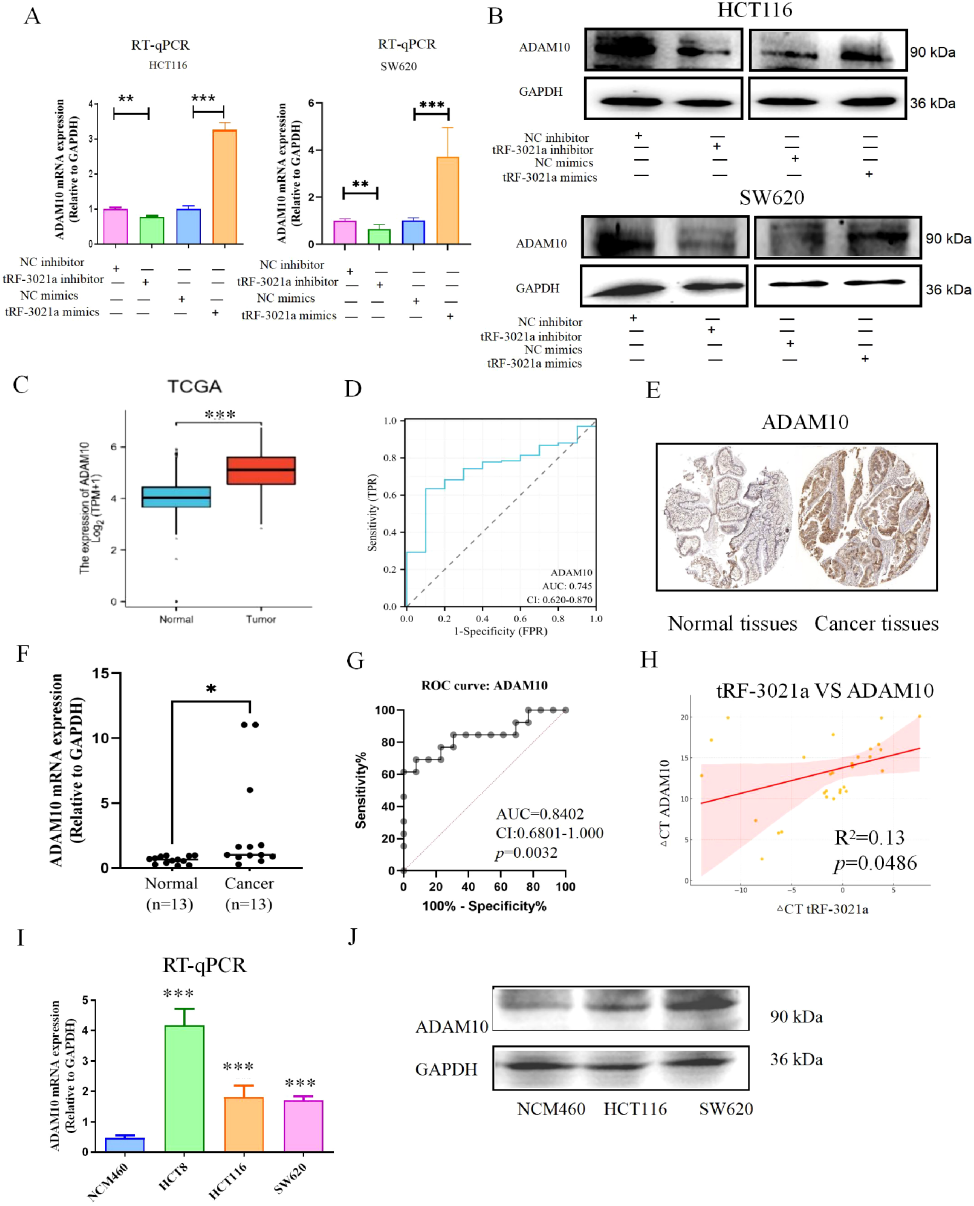
Mechanistic and clinical validation of tRF-3021a-mediated ADAM10 regulation in colorectal cancer. **(A)** RT-qPCR validation of ADAM10 transcriptional regulation by tRF-3021a modulators in CRC cells. **(B)** Western blot analysis confirming concordant protein-level changes. **(C)** TCGA-COAD dataset analysis showing ADAM10 upregulation in CRC tissues vs normal controls. **(D)** Diagnostic potential of ADAM10 with AUC=0.745 (95% CI:0.62-0.87) in TCGA cohort. **(E)** Representative IHC images from Human Protein Atlas (HPA ID: CAB000041) demonstrating ADAM10 overexpression in CRC specimens. **(F)** Validation cohort (n=13 pairs) showing 3.4-fold ADAM10 mRNA elevation in tumors. **(G)** Diagnostic accuracy in validation cohort. **(H)** Significant positive correlation between tRF-3021a and ADAM10 expression. **(I, J)** Consistent ADAM10 overexpression in CRC cell lines compared to normal NCM460 cells. *P < 0.05, **P < 0.01, and ***P < 0.001.

Given the pivotal role of the tRF-3021a/ADAM10 axis in impairing NK cell-mediated cytotoxicity, we further elucidated the molecular mechanism underlying tRF-3021a-dependent regulation of ADAM10. RT-qPCR and Western blot analyses in CRC cell lines (HCT116, SW620) demonstrated that tRF-3021a overexpression significantly upregulated both ADAM10 mRNA (*p*<0.01) and protein levels (*p*<0.05), whereas its knockdown elicited converse effects (**Figures 4A, B**). Notably, the tRF-3021a-mediated suppression of ADAM10 mRNA in HCT116 cells (*p*<0.001) corroborated our RNA-seq data (**Figure 3D**), validating the consistency across experimental platforms.

### 3.5 Clinical relevance of ADAM10 in CRC pathogenesis

Bioinformatic analysis of TCGA-COAD dataset revealed significant ADAM10 upregulation in CRC tissues compared to normal controls (*p*<0.001, **Figure 4C**), with ROC analysis demonstrating diagnostic potential (AUC=0.745, 95% CI:0.62-0.87, **Figure 4D**). Immunohistochemical validation via the Human Protein Atlas confirmed elevated ADAM10 protein expression in CRC specimens (HPA ID: CAB000041, **Figure 4E**). In our clinical cohort, ADAM10 mRNA was significantly upregulated in 13 tumor tissues (*p*<0.05, **Figure 4F**), exhibiting superior diagnostic accuracy (AUC=0.8402, CI:0.6801-1.000, *p*=0.0032, **Figure 4G**). Strikingly, ADAM10 expression positively correlated with tRF-3021a levels in patient tissues (Pearson R^2^ = 0.13, *p*=0.0486, **Figure 4H**). Comparative analysis of CRC cell lines (HCT116, HCT8, SW620) versus normal colon epithelial NCM460 cells revealed consistent ADAM10 overexpression at both mRNA (*p*<0.001) and protein levels (**Figures 4I, J**).

As a member of the adamalysin metalloproteinase family, ADAM10 has been mechanistically linked to inflammation-driven carcinogenesis through proteolytic shedding of membrane-anchored ligands (26). Our findings align with prior reports of its oncogenic roles in gastric and hepatocellular carcinomas (27, 28), while newly establishing its diagnostic utility in CRC (28). The tRF-3021a/ADAM10 regulatory axis may represent a therapeutic target to restore NK cell immunosurveillance, potentially via inhibition of MICA shedding.

### 3.6 tRF-3021a/ADAM10 axis promotes immunoevasion in colorectal cancer by cleaving MICA to suppress antitumor immunity

**Figure 5:**
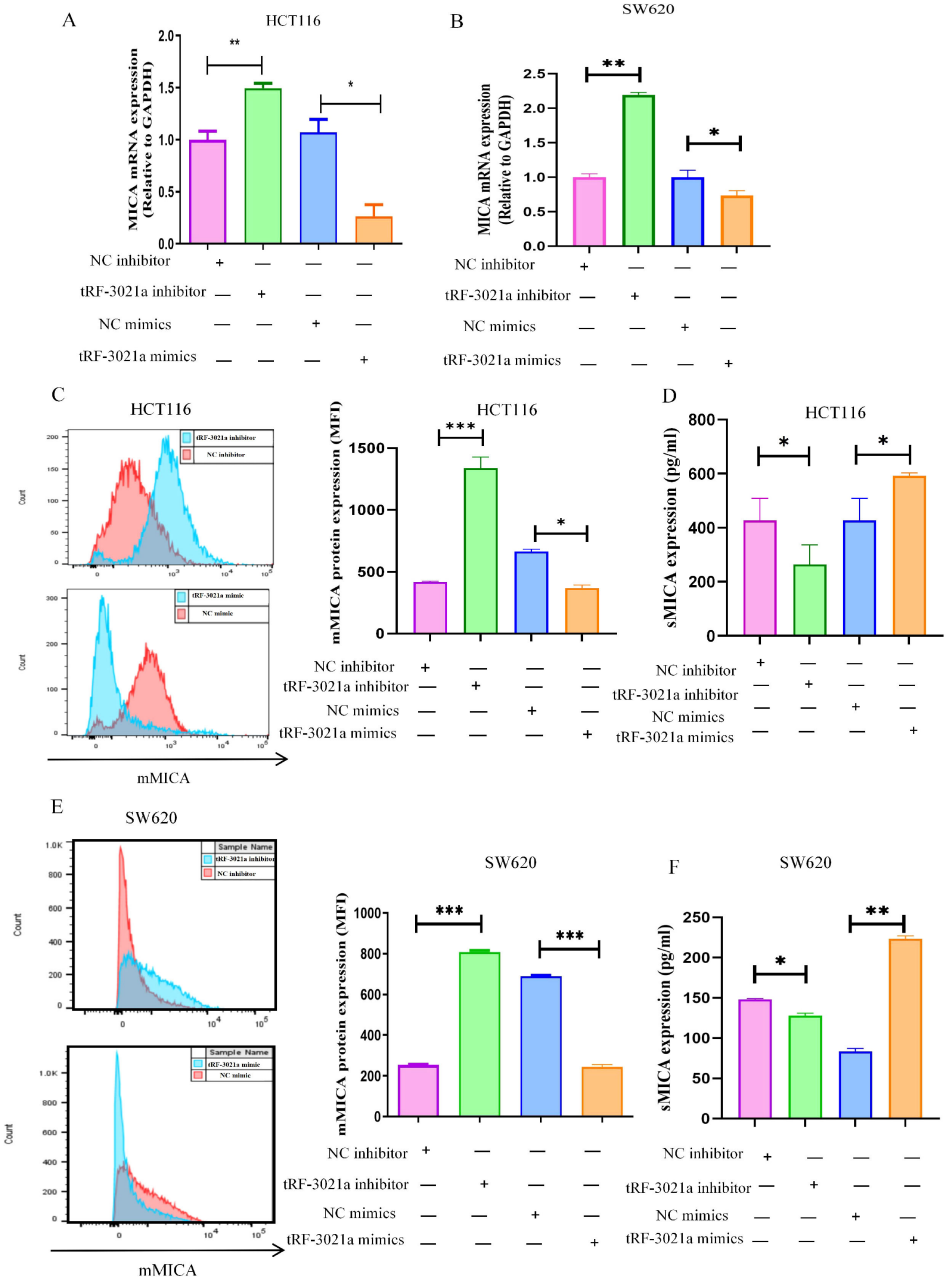
Regulation of MICA expression by tRF-3021a. **(A, B)** RT-qPCR analysis demonstrated that tRF-3021a downregulated MICA mRNA expression. **(C, E)** Flow cytometry analysis was conducted to assess the expression of MICA on the cell membrane. **(D, F)** ELISA results revealed a positive correlation between tRF-3021a and soluble MICA (sMICA) expression. *P < 0.05, **P < 0.01, and ***P < 0.001.

We subsequently examined the potential impact of tRF-3021a on MICA mRNA and protein expression. In HCT116 cells, the upregulation of MICA mRNA following tRF-3021a interference was consistent with the RNA sequencing data. Specifically, in colorectal cancer cell lines, MICA mRNA expression was elevated in the tRF-3021a interference group, whereas it was suppressed in the tRF-3021a overexpression group (**Figures 5A, B**). To assess the regulation of membrane-bound MICA (mMICA) by tRF-3021a, flow cytometry analysis was performed. The results showed an upregulation of mMICA expression in the tRF-3021a interference group and a downregulation in the overexpression group (**Figures 5C, E**). Notably, ELISA analysis of the colorectal cancer cell supernatants revealed an inverse relationship between the expression of soluble MICA (sMICA) and mMICA. Specifically, sMICA levels were decreased in the tRF-3021a interference group and increased in the overexpression group (**Figures 5D, F**). Previous studies have indicated that ADAM10 can cleave mMICA to generate sMICA, thereby promoting tumorigenesis in colorectal cancer. Our findings suggest that tRF-3021a overexpression increases ADAM10 expression, leading to a reduction in mMICA and an elevation in sMICA production. This, in turn, facilitates colorectal cancer progression through an NKG2D receptor-mediated immune escape mechanism, resulting in the inhibition of NK cell activation by promoting MICA shedding via ADAM10.

**Figure 6:**
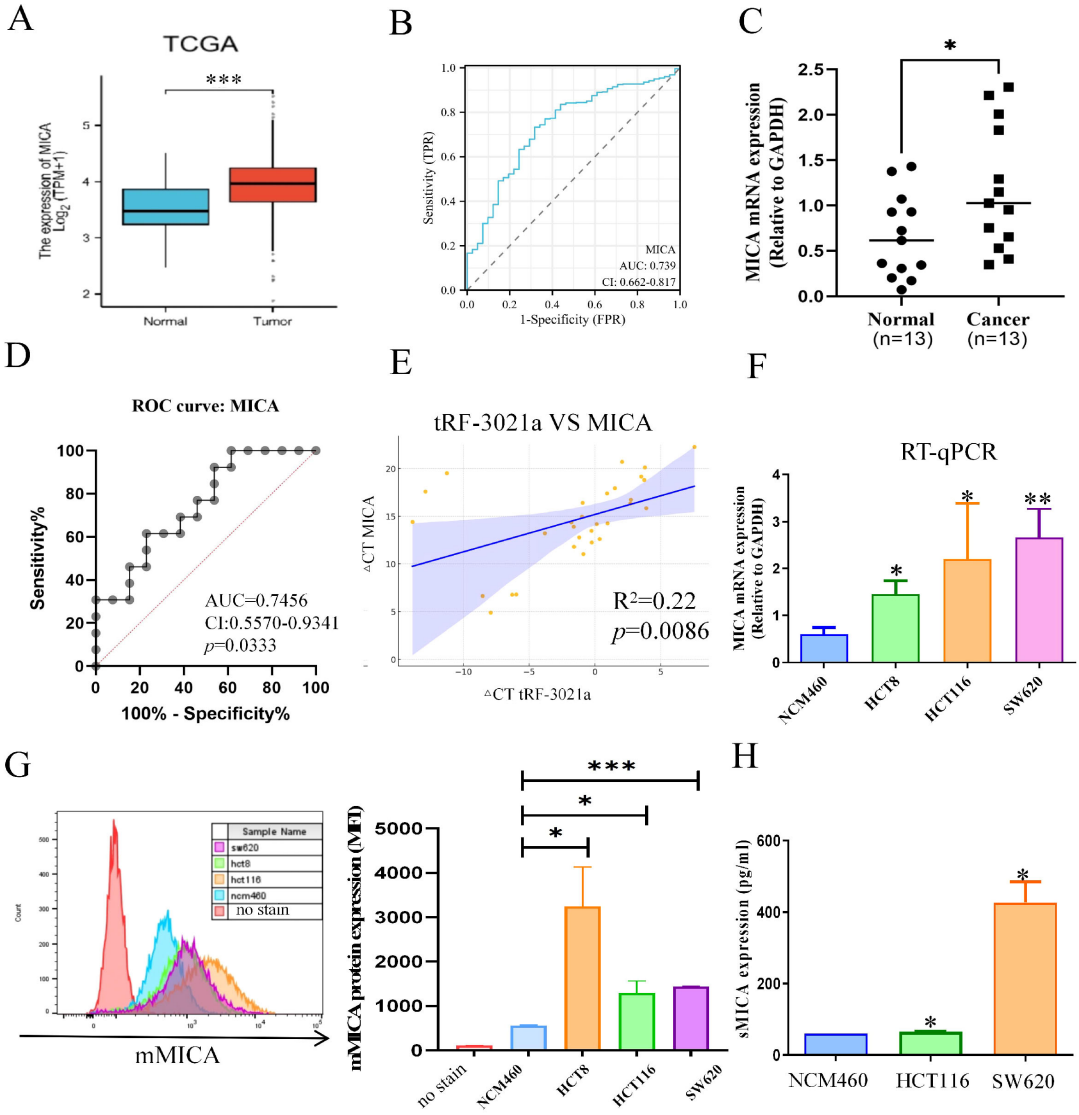
The relationship between MICA and the clinicopathological characteristics of CRC patients. **(A)** Based on TCGA database, MICA mRNA expression was higher in CRC tissues than normal tissues. **(B)** ROC curve to evaluate the relationship between MICA and CRC growth. **(C)** Based on the CRC patient’s cohort, MICA was significantly upregulated in CRC tissues. **(D)** ROC analysis of MICA in CRC patient’s cohort. **(E)** Graphs illustrate the positive correlation between tRF-3021a and MICA expression in CRC patients, derived from the CRC patient’s cohort. **(F)** RT-qPCR analysis of MICA mRNA expression levels in CRC cell lines and normal colon cells (NCM460). **(G)** Flow cytometry analysis of mMICA protein levels in CRC cell lines and normal colon cells. **(H)** ELISAs revealed that sMICA protein expression was upregulated in CRC cell lines. *P < 0.05, **P < 0.01, and ***P < 0.001.

Furthermore, we analyzed MICA expression using the TCGA database and experimental validation, as the role of MICA in colorectal cancer remains underexplored. Notably, the expression level of MICA was significantly higher in colorectal cancer tissues compared to normal tissues, as observed in the TCGA database (**Figure 6A**). ROC analysis revealed an AUC of 0.739 with a confidence interval (CI) of 0.662-0.817 (**Figure 6B**). To further validate these findings, RT-qPCR was performed on 13 pairs of colorectal cancer tissues and adjacent normal tissues, yielding results similar to those from the TCGA dataset (**Figure 6C**). MICA mRNA expression was significantly higher in colorectal cancer tissues compared to normal tissues, and ROC analysis indicated an AUC of 0.7456, with a CI of 0.556-0.9341, and a *p*-value of 0.0333 (**Figure 6D**). Additionally, our analysis showed a positive correlation between tRF-3021a and MICA expression, with an R² value of 0.22 and a *p*-value of 0.0086 (**Figure 6E**). Moreover, both MICA mRNA and protein expression were significantly elevated in colorectal cancer cell lines (**Figures 6F–H**). These results suggest that MICA could serve as a potential biomarker in colorectal cancer. Importantly, the abundant expression of mMICA in colorectal cancer cells may contribute to the substantial production of sMICA, which plays a critical role in immune escape by NK cells.

### 3.7 tRF-3021a promotes proliferation, inhibits CRC cells apoptosis, and results to immune escape of CRC

**Figure 7:**
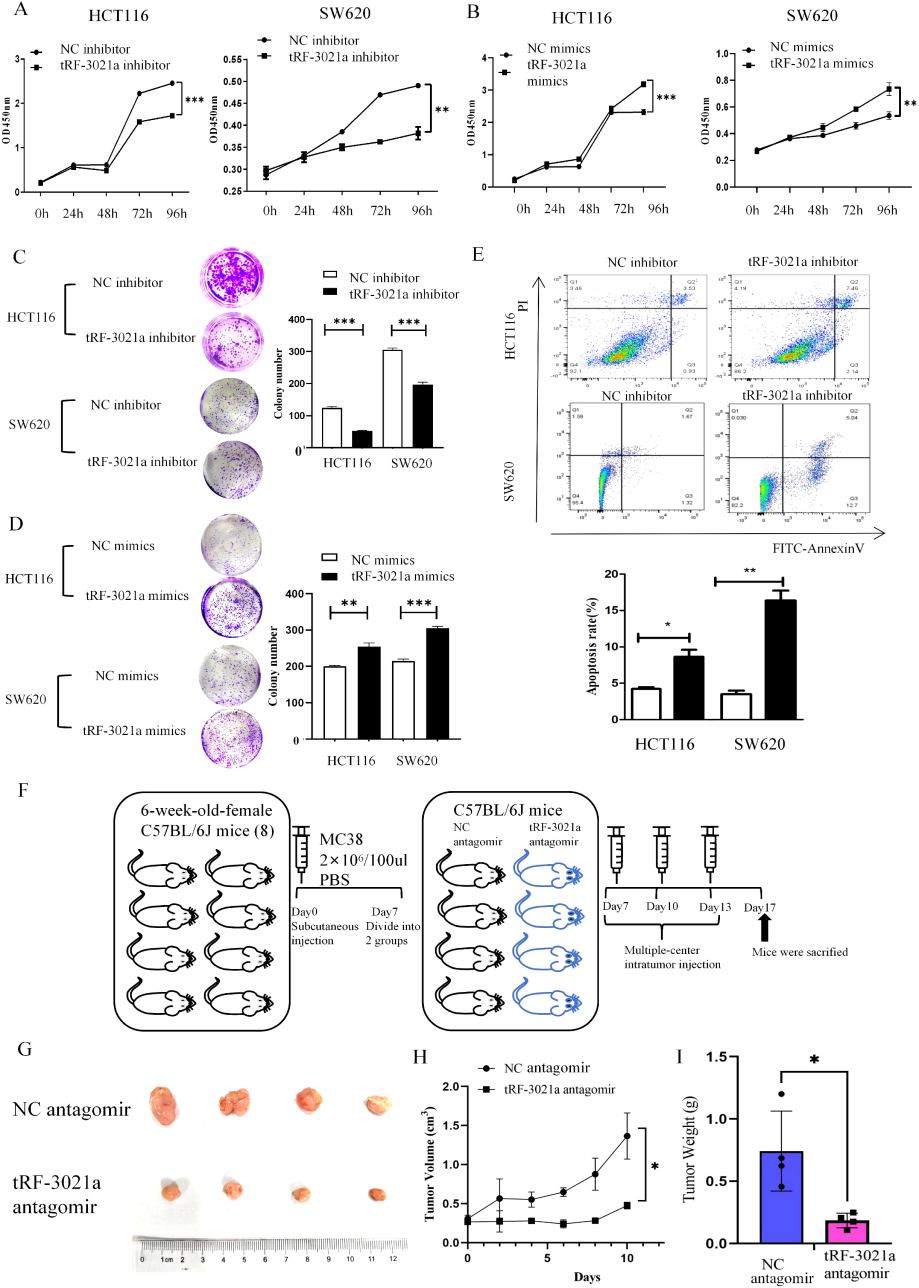
tRF-3021a promoted proliferation, colony formation and inhibited cell apoptosis in CRC cells *in vitro* and murine models of CRC in vivio. **(A, B) **CCK8 assay and **(C, D)** colony formation was performed to evaluated proliferation capacity after overexpression and inhibition of tRF-3021a in CRC cells. **(E)** Cell apoptosis analysis showed that tRF-3021a has affected the cell apoptosis of CRC cell lines. **(F)** Schematic diagram of animal experiments. **(G)** Representative tumor images (n=4 for each group). **(H)** Tumor volume measured 2, 4, 6, 8, 10 days after MC38 cell injection. **(I)** Tumor weight measured 10 days after MC38 cell injection (n=4 for each group). *P < 0.05, **P < 0.01, and ***P < 0.001.

Building on the observed regulatory role of tRF-3021a in upregulating ADAM10 and sMICA expression in colorectal cancer (CRC) cells—where ADAM10-mediated cleavage of mMICA generates sMICA to promote tumor growth by evading NK cell immune surveillance—we further investigated the functional impact of tRF-3021a on CRC malignancy. To determine whether tRF-3021a directly drives oncogenic progression, we conducted systematic gain- and loss-of-function experiments *in vitro*. Cell proliferation assays using CCK-8 revealed that tRF-3021a knockdown significantly suppressed the proliferative capacity of CRC cell lines (**Figure 7A**), whereas its markedly overexpression enhanced proliferation (**Figure 7B**). Consistent with these findings, clonogenic assays demonstrated that tRF-3021a depletion substantially reduced colony-forming ability (**Figure 7C**), while its overexpression potentiated clonogenicity (**Figure 7D**). Notably, tRF-3021a silencing also induced apoptosis in CRC cells (**Figure 7E**), underscoring its role in sustaining cell survival. In our investigation of tRF-3021a’s role in antitumor immunity, immune-competent female C57BL/6J mice bearing MC38 cell-derived allograft demonstrated significant tumor suppression following tRF-3021a antagomir administration (**Figure 7F**). Comparative analysis at 17 days post-implantation revealed markedly reduced tumor volume (*p* < 0.05) and decreased mass (*p* < 0.01) in the treatment group compared to controls, as illustrated in **Figures 7G–I**.

These results collectively establish tRF-3021a as a critical oncogenic driver in CRC, potentiating tumor progression through dual mechanisms: enhancing proliferative and suppressing apoptosis. The observed functional synergy between tRF-3021a, ADAM10, and sMICA suggests a coordinated axis that facilitates immune evasion and tumor growth, positioning tRF-3021a as a potential therapeutic target for disrupting this pathogenic cascade.

## 4 Discussion

The progression of colorectal cancer often begins with micro-metastasis (25). Although micro-metastatic cells in bone marrow, that is, diffuse tumor cells, have independent prognostic value for patients with colorectal cancer, invasive puncture is required (25). The limitations of current clinical diagnostic tools in predicting metastasis progression and detecting minimal residual lesions are major barriers to improving prognosis (29). Despite advances in cancer research and treatment, cancers that are difficult to detect early and easy to metastasize late remain the leading causes of cancer-related death worldwide (30). If the micro-metastasis status of colorectal cancer patients can be accurately detected in the early stage, it will help to locate and find the high-risk groups of recurrence and metastasis in the early stage, which is of great significance to strengthen patient follow-up, take corresponding treatment measures, reduce tumor recurrence and metastasis and improve the survival rate of patients (31). Tumor Marker (TM) usually refers to a class of substances, including proteins, hormones and enzymes, that are produced by tumor cells themselves or abnormally produced and elevated by the body in response to tumor cells during the occurrence and proliferation of malignant tumors, reflecting the existence and growth of tumors and monitoring the response of tumors to treatment (32). The more vigorous the tumor growth, the more corresponding markers (33). Conversely, tumor growth is inhibited and the number of markers produced is also reduced. Tumor cells are difficult to detect in the early stages of development in the body, only when the tumor cells secrete specific protein enrichment, can be detected in the blood, this “time difference” may be three or four months (33). In addition, if some tumors do not secrete the type of protein detected, it is more difficult to “catch”. Therefore, dynamic observation and sensitive and effective tumor markers (ncRNAs) are very important (24). “Liquid biopsy” tsRNA specifically expressed in peripheral blood or exosomes of patients has relatively stable structure and function, specific production and enrichment, and is easy to detect (33). It is not only minimally invasive, but also a living specimen carrying rich disease information, which can accurately present the current status of tumors and provide a diagnostic basis for the treatment of colorectal cancer (34). It may be used as an effective tumor marker to provide a highly specific, highly sensitive, real-time and minimally invasive method for early detection, prognosis and prediction of anti-cancer drug response, so as to realize its full potential as a high-accuracy and predictive tool for early detection and anti-metastasis therapy, which has important clinical significance, with both opportunities and challenges (32). The content of 5’ tRF-GlyGCC in the plasma of colorectal cancer patients was significantly higher than that of healthy patients. Based on ROC analysis, the AUC of 5’ tRF-GlyGCC in CRC group was 0.882. Blood cells co-cultured with colorectal cancer cells or mice xenografted with colorectal cancer tumors showed elevated 5’ tRF-GlyGCC. Increased expression of 5’ tRF-GlyGCC is dependent on upregulation of AlkB homolog3 (ALKBH3), a tRNA demethylase that promotes tRNA cleavage to tRF (34). Plasma 5’ tRF-GlyGCC levels are a promising biomarker for the diagnosis of CRC (34). 5’ tiRNA-Gly-GCC is upregulated and regulated through N (7)-methylguanosine tRNA modification mediated by METTL1. 5’ tiRNA-Gly-GCC regulates the JAK1/STAT6 signaling pathway by targeting SPIB. The delivery of synthetic poly (β-amino ester) combined with 5-FU and tiRNA-Gly-GCC inhibitors effectively suppresses tumor growth, enhances CRC sensitivity to 5-FU, and shows no significant adverse reactions in subcutaneous tumors, potentially providing a promising nanotherapy strategy for treating 5-FU resistant CRC (20). Reduced expression of tRF3008A in colorectal cancer is significantly associated with advanced and metastatic disease of colorectal cancer. tRF3008A is an independent prognostic biomarker for CRC. Functionally, tRF3008A inhibits CRC by inhibiting endogenous FOXK1 and is a regulator of proliferation and migration of the Wnt/β-catenin pathway *in vivo* and *in vitro*. tRF3008A binds to the AGO protein and targets FOXK1 mRNA (35). tRF-3022b is upregulated in colorectal cancer cells and secreted by exosomes. Silencing tRF-3022b can promote the polarization of M2 macrophages. tRF-3022b binds to the effects of lectin 1 (LGALS1) and macrophage migration inhibitor (MIF) in colorectal cancer cells, and by regulating MIF in M2 macrophages. tRF-3022b, which may affect colorectal cancer tumor growth and polarization of M2 macrophages by binding LGALS1 and MIF (36).

However, most of the evidence comes from small-sample studies. The dynamic change patterns of tsRNA in response to treatment or as a predictor of recurrence have not yet been established, and there is a lack of large-scale cohort validation. Commercial miRNA detection kits are already available, while tsRNA is still in the research stage. This will motivate us to further advance the research on tsRNA. Since MICA is mainly expressed in human cells and does not exist in mouse cells, in animal experiments, more methods need to be employed for research, such as using immune-deficient mice or humanized mice. The mechanism of this animal model needs to be further explored in the future.

**Figure 8:**
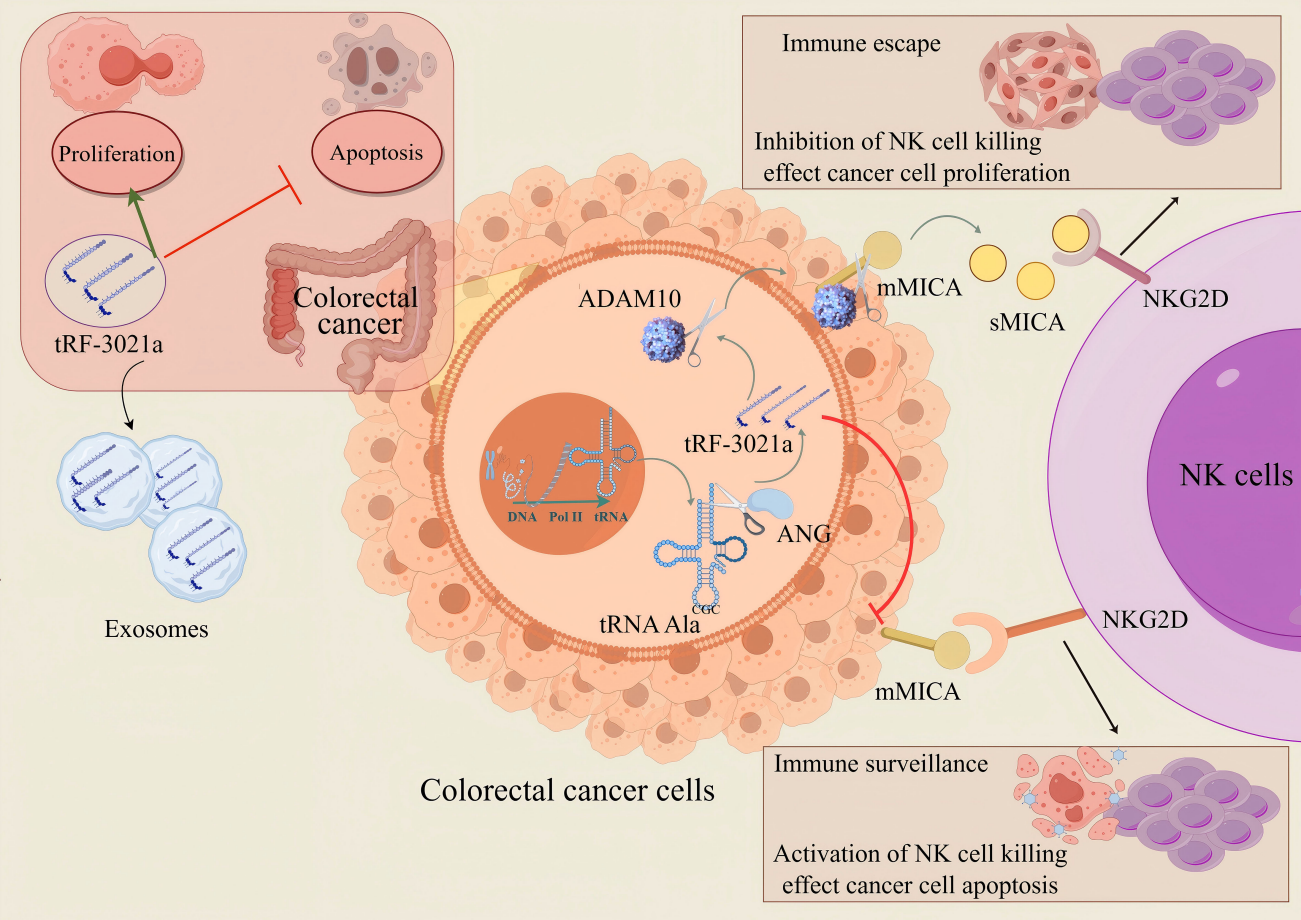
The proposed model tRF-3021a may activate the immune escape mechanism of tumor cells against NK cells by up-regulating the expression of ADAM10 protein, down-regulating the expression of mMICA protein and enhancing the expression of soluble MICA. (The red T-shaped arrows represent inhibitory effects, while the green arrows represent promoting effects.).

Notably, tumor microenvironment analysis proposed a critical mechanism: In CRC cells, membrane-bound MICA (mMICA) is cleaved and hydrolyzed by the ADAMs family, which may drive the proteolytic shedding and generate a large amount of soluble MICA (sMICA). This molecular transformation may be the basis for the observed increase in sMICA levels. sMICA promotes immune evasion by competitively binding to the NKG2D receptor on NK cells, thereby impairing their cytotoxic activity. Our findings not only highlight tRF-3021a as a promising diagnostic biomarker for CRC but also provide mechanistic insights for developing immunotherapeutic strategies targeting the MICA/NKG2D axis. tRF-3021a is upregulated in CRC tissues, exosomes and cells and promotes CRC cells proliferation, colony formation and inhibitor apoptosis, which is cleaved by ANG. tRF-3021a is highly expressed in colorectal cancer, and by upregulating ADAM10 protein expression, cleave membrane MICA forms more soluble MICA, blocking the cytotoxic effect of NKG2D activated receptors in NK cells, and ultimately leading to immune escape in colorectal cancer (**Figure 8**). Design suppressor drugs targeting tRF-3021a to down-regulate the expression of ADAM10 protein, maintain the expression of membrane protein MICA, reduce soluble MICA, and remove the immune escape of tumor cells to NK cells. Therefore, drug development targeting tRNA derivative (tRF-3021a) provides a new idea for tumor immunotherapy.

## 5 Conclusions

3’ tRF-Ala-CGC/tRF-3021a is novel and non-invasive CRC biomarker and promotes CRC cells proliferation, colony formation and inhibitor apoptosis, which also suppressed NK cell immune surveillance by promoting MICA shedding, which serves as a potential therapeutic target for CRC.

## Data Availability

The datasets presented in this study can be found in online repositories. The names of the repository/repositories and accession number(s) can be found in the article/**Supplementary Material**.
