# Supplementary material for: tRNA-derived small RNA 3’ tRF-Ala CGC obstructs NK cytotoxicity via cleavage of membrane protein MICA in colorectal cancer
Source: Front Immunol. 2025 Jul 14;16:1620550. doi: 10.3389/fimmu.2025.1620550 (PMC12301416; doi:10.3389/fimmu.2025.1620550)
Supplement: Supplementary file 1 [file DataSheet1.docx]

**Supplementary Table 1.** **Primers used for real-time PCR assay.**

| **Gene** | **Forward (5'-3')** | **Reverse (5'-3')** |
| --- | --- | --- |
| U6 | CTCGCTTCGGCAGCACA | AACGCTTCACGAATTTGCGT |
| tRF-3021a | GCGATATCCCCGGCATCT | AGTGCAGGGTCCGAGGTATT |
| GAPDH | GCACCGTCAAGGCTGAGAAC | TGGTGAAGACGCCAGTGGA |
| ANG | CAAGGCCATCTGTGAAAACAAG | CAGGGGGAACCTCCATGTAG |
| ADAM10 | CTGGCCAACCTATTTGTGGAA | GACCTTGACTTGGACTGCACTG |
| MICA | CACAGCGGGAATCACAGCACTC | ATAGCAGCAGCAGCAACAGCAG |
| tRF-3021a stem loop primer | GTCGTATCCAGTGCAGGGTCCGAGGTATTCGCACTGGATACGACTGGTGGAG | |

**Supplementary Table 2. Small interfering RNA (siRNA) and mimic sequences.**

| Gene | Sequence (5'-3') |
| --- | --- |
| NC siRNA | UUCUCCGAACGUGUCACGUTT |
| ANG siRNA | CCUGACCCAGCACUAUGAUGCCAAA |
| tRF-3021a inhibitor | TGGTGGAGATGCCGGGGA |
| tRF-3021a mimic | TCCCCGGCATCTCCACCA |

**
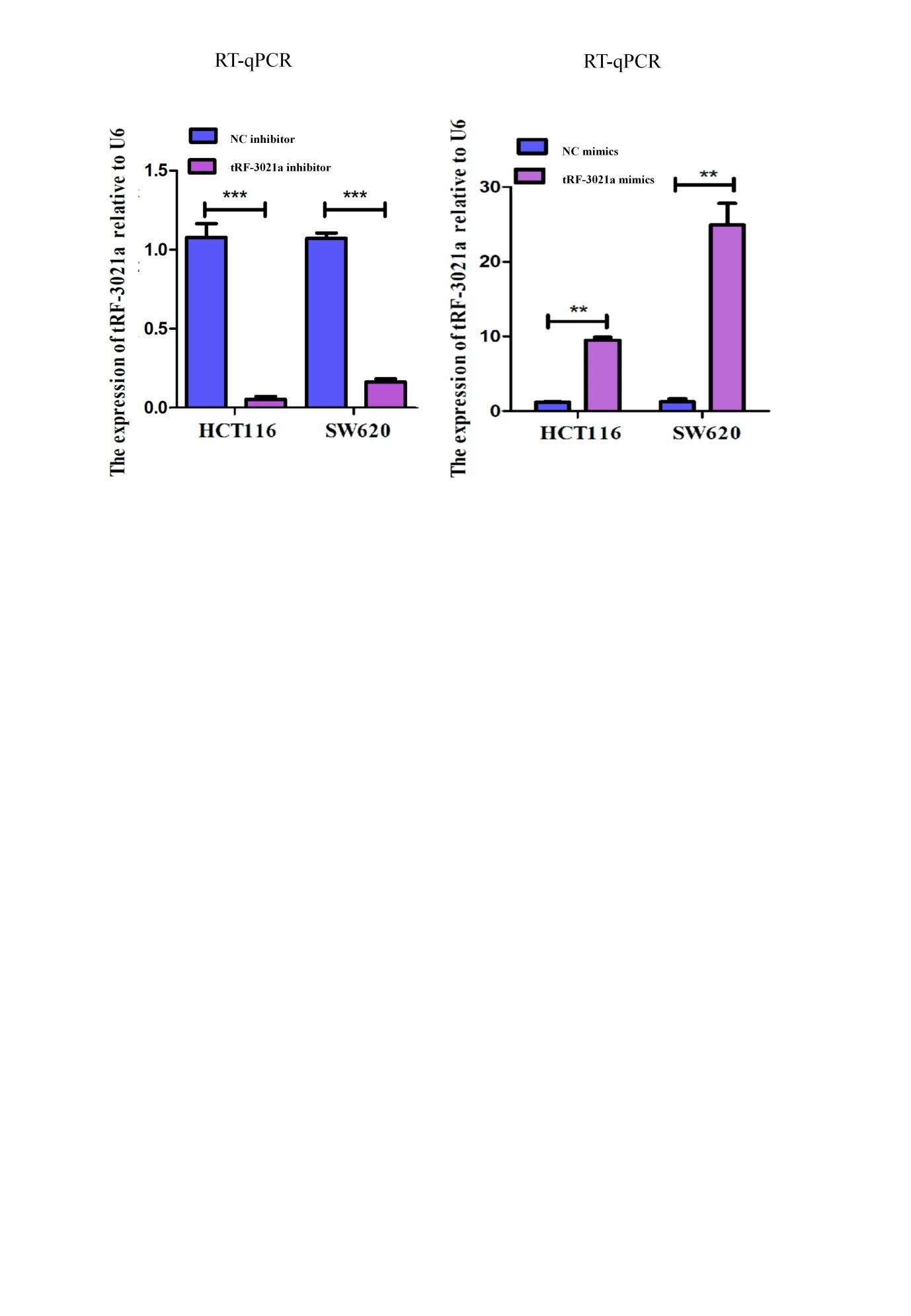
**

**Figure S1. qRT-PCR validation of different genes.** The expression level of tRF-3021a in the colorectal cells after transfection of the tRF-3021a mimic and inhibitor.
